# Molecular Effect of Variants in Serotonin Transporter Gene in Women with Alcohol Use Disorder

**DOI:** 10.3390/cells14100699

**Published:** 2025-05-12

**Authors:** Monika Rychel, Aleksandra Suchanecka, Jolanta Chmielowiec, Krzysztof Chmielowiec, Jacek Różański, Jolanta Masiak, Anna Grzywacz, Agnieszka Boroń

**Affiliations:** 1 Department of Clinical and Molecular Biochemistry, Pomeranian Medical University in Szczecin, Powstańców Wielkopolskich 72 St., 70-111 Szczecin, Poland; monika.rychel@gmail.com (M.R.); agnieszka.boron@pum.edu.pl (A.B.); 2 Independent Laboratory of Behavioral Genetics and Epigenetics, Pomeranian Medical University in Szczecin, Powstańców Wielkopolskich 72 St., 70-111 Szczecin, Poland; aleksandra.suchanecka@pum.edu.pl; 3 Department of Hygiene and Epidemiology, Collegium Medicum, University of Zielona Góra, 28 Zyty St., 65-046 Zielona Góra, Poland; chmiele1@o2.pl (J.C.); chmiele@vp.pl (K.C.); 4 Department of Nephrology, Transplantology and Internal Medicine, Pomeranian Medical University in Szczecin, Powstańców Wielkopolskich 72 St., 70-111 Szczecin, Poland; jacekrozanski@wp.pl; 5 Second Department of Psychiatry and Psychiatric Rehabilitation, Medical University of Lublin, Głuska 1 St., 20-059 Lublin, Poland; jolanta.masiak@umlub.pl; 6 Department of Molecular Biology, Gdansk University of Physical Education and Sport, Kazimierza Górskiego 1 St., 80-336 Gdansk, Poland

**Keywords:** *5-HTTLPR*, neuroticism, alcohol use disorder, NEO-FFI, STAI, women, *SLC6A4*, personality traits

## Abstract

The dysregulation of the serotonin system has been implicated in the pathophysiology of alcohol use disorders. Meta-analytic evidence suggests a significant correlation between genetic variation in the serotonin transporter gene and the risk of alcohol dependence. Hence, we aimed to analyse the association between *5-HTTLPR* polymorphism and alcohol use disorder in a group of women and to perform an interaction analysis of *5-HTTLPR* variants, personality traits, and AUD. The study group comprised 213 female volunteers; 101 were diagnosed with alcohol addiction, and 112 were not dependent on any substance or behaviour. The *5-HTTLPR* variants were identified by PCR, and the resulting products were separated electrophoretically. When comparing the AUD group with the controls, we observed significant differences in the distribution of *5-HTTLPR* genotypes (*p* = 0.0230) and alleles (*p* = 0.0046). We also observed a significant impact of the *5-HTTLPR* genotype (*p* = 0.0001) on the Neuroticism and Extraversion (*p* = 0.0037) scales. Additionally, there was a statistically significant impact of *5-HTTLPR* genotype interaction and alcohol dependency or lack of it on the Neuroticism scale (*p* < 0.0001). The observed interaction suggests that the effect of the *5-HTTLPR* on neuroticism may be exacerbated or attenuated in the presence of alcohol addiction. Further investigation is needed to elucidate the precise nature of this interaction. Still, it potentially indicates a gene–environment interaction where the genetic predisposition conferred by the *5-HTTLPR* polymorphism interacts with the environmental stressor of alcohol dependence to influence neuroticism.

## 1. Introduction

Alcohol is globally one of the most commonly used psychoactive substances [1]. Alcohol misuse is associated with significant health concerns. In the short term, alcohol misuse significantly increases the risk of injuries from accidents, aggression, and violence, and excessive consumption potentially leads to fatal alcohol poisoning or trauma-related mortality. Long-term alcohol abuse is strongly associated with the occurrence of mental health disorders, i.e., depression, anxiety, and alcohol-induced psychosis, as well as a range of physical comorbidities, such as liver disease, various carcinomas, and fetal alcohol spectrum disorders during pregnancy [1].

Clinically, alcohol misuse—particularly when it progresses to alcohol use disorder (AUD) or dependence (AD)—has profound behavioural and physiological consequences. Globally, an estimated 400 million people (7% of the population) aged over 15 years live with AUD, while 209 million (3.7% of adults) suffer from alcohol dependence [2]. In 2019 alone, alcohol misuse was responsible for 2.6 million deaths (4.7% of all global deaths). It accounted for 115.9 million disability-adjusted life years (DALYs), primarily due to injuries, non-communicable diseases, and mental and substance use disorders. Of these, 89.5 million DALYs were attributable to premature mortality (years of life lost—YLL; 5.3% of total YLL), and 26.4 million were due to morbidity (years lived with disability—YLD; 3.2% of total YLD). These statistics [2] underscore the substantial behavioural, clinical, and economic burden imposed by alcohol misuse on individuals and societies worldwide.

Understanding the detrimental effects of alcohol is challenging, in part due to the inconsistencies in definitions and diagnostic classifications. The definition of a standard alcohol unit and the recommended guidelines for consumption vary considerably across different nations [3]. Furthermore, the *Diagnostic and Statistical Manual of Mental Disorders*, 5th Edition (DSM-5) [4] and the *International Classification of Diseases*, 11th Revision (ICD-11) [5] employ notably different systems for categorising clinically relevant alcohol usage. The DSM-5 utilises a single diagnostic category, Alcohol Use Disorder (AUD), with three levels of severity: mild, moderate, and severe. In contrast, the ICD-11 employs two diagnostic categories that reflect increasing severity: ‘harmful pattern of use of alcohol’ followed by ‘alcohol dependence,’ and also includes a subclinical risk factor designation of ‘hazardous alcohol use’. Despite the presented differences in formal classification, both diagnostic systems fundamentally reflect an impaired capacity to regulate alcohol consumption.

In the mechanics of the development of alcohol dependence, it is important to understand the neurobiological basis behind this process. Alcohol, due to its simple molecular structure, is able to penetrate the blood–brain barrier and thus affect neurotransmitter systems by modulating various receptors and transporters [6]. In particular, the dopaminergic (rewarding system) and serotonergic systems play an important role. The most important neurotransmitter of the rewarding system is dopamine (DA, 3,4-dihydroxytyramine) synthesized from tyrosine in the ventral tegmental area (VTA) and in the substantia nigra (SN) [7]. The regulation of the amount of dopamine is carried out by five D1-5 receptors belonging to the GPCR superfamily, located in the outer cell membranes of the cells receiving the signal [8]. In the serotonergic system, the main role is played by a monoamine neurotransmitter —serotonin (5-HT, 5-hydroxytryptamine), synthesized from tryptophan in neurons of the middle (MRN) and dorsal raphe nuclei (DRN) of the brainstem. The amount of serotonin in synaptic spaces is controlled by seven 5-HT1-7 transmembrane receptors [9]. Both systems work together in the functioning of the reward system. Anatomically, serotonin DRN neurons are linked to dopamine VTA neurons for the innervation of the same areas. In addition, many different 5-HT receptors are scattered throughout the dopaminergic system to modulate 5-HT signals, which consequently affects dopamine expression since for 5-HTR3A within VTA 80% of DA neurons are positive. Although the two systems have previously been classified as opposite, significant interactions between them have been proven in recent years. In this regard, the existence of coordinated pre- and post-synaptic neuronal activity between DA and 5-HT has been found to ensure continuous synaptic plasticity to enhance the reward system and allow behavioural flexibility. In this, it has been proven that reward signals activate (in the same way as DA neurons) DRN 5-HT neurons [10].

In the reward system, three peripheral neural pathways can be distinguished as follows: the mesolimbic pathway (the process of learning, addiction, motivation, perception of pleasure), the nigrostriatal pathway (control and cognition of motor functions), and the mesocortical pathway (decision making and executive skills), which are controlled by the VTA and SN mDAneric neurons [11]. Alcohol can significantly affect the mesolimbic pathway, the nucleus accumbens (NAC), and the ventral tegmental area by modulating glutamatergic, dopaminergic, and serotonergic neurotransmission. It is hypothesised that continuous alcohol consumption alters the level of sensitivity of the DA receptor, thereby reducing the intensity of the reward response. This consequently leads to an increase in compensatory drinking attempts [6,12]. Alcohol directly affects the dopaminergic pathway by limiting the function of the monoamine oxidase protein, i.e., DA breakdown. A prolonged response to an increased amount of dopamine in the postsynaptic space results in a continuous feeling of pleasure. A disturbed functioning of the reward system can contribute to addiction [13]. Studies have also shown that alcohol can indirectly increase the amount of dopamine in the nucleus accumbens by modulating GABAergic neurons by interfering with the ability to produce neuronal action potential, e.g., by affecting the serotonin 5-HT receptor [13]. Serotonin also has a modulating effect on the reward system due to the innervation of the limbic–corticostriatal circuit. Violent alcohol consumption contributes to an increase in 5-HT concentrations in extracellular spaces and changes the functioning of 5-HTR [9]. The serotonergic system may influence the function of other ligands, including dopamine, through the action of the 5-HT3 receptor on DA release in the mesolimbic pathway [9,14]. Recent studies illustrate the direct effect of 5-HT on the reward system through the mesolimbic pathway on NAC and VTA [15]. Ethanol increases the secretion of serotonin to the NAC (including the innervation of the NAC shell and the medulla) thereby intensifying the reward system response. In addition, a study on mice (exposed to the continuous effects of ethanol) showed the effect of 5-HT2C receptor signalling in the NAC coating on the escalation of alcohol dependence [16]. Although the mechanisms behind the neurobiological background of addiction are very extensive, they are still insufficiently studied and many processes have not yet been elucidated. However, the dopaminergic and serotonergic systems can be modulated by alcohol and affect each other.

Dysregulation of the serotonin system has been associated with the pathophysiology of alcohol use disorders. Cloninger [17] proposed that deficits in serotonergic transmission are linked with early-onset alcohol dependency and addictive behaviours. Serotonin, a monoamine neurotransmitter, is crucial in modulating a wide array of functions, including sleep, mood, food intake, vascular tone, pain, behaviour, motor activity, and platelet activity [18,19,20]. Additionally, serotonin is associated with a complex novelty-seeking trait which is sometimes associated with increased risk-taking behaviour [21]. Synthesised in the raphe nuclei, serotonin is transported to brain regions—the subcortical areas, cerebral cortex, and hippocampus—influencing reward pathways. After release into the synaptic cleft, serotonin interacts with postsynaptic receptors, initiating neuronal signalling. The subsequent reuptake of serotonin via serotonin transporters (5-HTT) on presynaptic neurons and enzymatic degradation by monoamine oxidase [22] regulate the magnitude and duration of these signals. Alterations in these processes are believed to contribute to various behavioural and neurocognitive dysfunctions, potentially predisposing individuals to alcohol use disorders, i.e., increased impulsivity, heightened negative mood, intensified alcohol craving, and altered responses to alcohol intake [23,24]. Additionally, acute alcohol usage results in an increase in the extracellular serotonin levels, while in chronic alcohol users decreased levels of 5-hydroxyindoleacetic acid (5-HIAA), the metabolite of serotonin, were found, indicating decreased 5-HT transmission [revised by [25]]. Global estimates regarding substance use indicates that 2.5 billion people drink alcohol [2] and around 1.25 billion people smoke tobacco (WHO, 2021) with a significant overlap between the two groups [26] and well-known comorbidities between alcohol, nicotine, and other substance dependency [27]. The usage of nicotine is associated with differential serotonin transporter availability [28] depending on the *5-HTTLPR* genotype, indicating the complex relationship between serotonin neurotransmission and psychoactive substances usage and dependency. Genetic factors are significantly involved in the aetiology of Alcohol Use Disorder (AUD), as evidenced by a body of research utilising diverse methodologies. Adoption studies have consistently demonstrated an elevated risk of developing alcohol use disorder among adopted individuals who have a positive biological family history of the disorder [29,30]. Twin studies further support this conclusion, revealing a higher concordance rate for AUDs in monozygotic twins compared with dizygotic twins [31,32]. The estimated heritability of AUD falls within 40–60% [32]. Furthermore, genetic influences extend beyond AUD, contributing to the pathophysiology of other substance use and psychiatric disorders, which share varying degrees of genetic risk with alcohol use disorders [33,34,35,36]. Notably, there is increasing evidence that the genetic predisposition to AUDs overlaps with the genetic predisposition to substance use disorders in general, as well as externalising psychopathology [37,38,39,40]. This suggests that genetic contributions to drinking behaviours encompass both alcohol-specific components, associated with the pharmacological effects of alcohol, and non-alcohol-specific components, related to broader behavioural patterns associated with over-consumption and impaired self-control.

The serotonin transporter (5-HTT), encoded by the *SLC6A4* gene located on chromosome 17q12, is crucial in regulating serotoninergic neurotransmission. The gene spans approximately 31 kilobases, encompasses 14 exons, and contains a functionally relevant polymorphic region in its promoter known as the 5-HT transporter-linked promoter region (*5-HTTLPR*) [41,42]. This polymorphic region consists of a guanine-cytosine (GC)-rich area with 20–23 base pair repeats and a 43-base pair insertion/deletion polymorphism. The common allelic variants, designated as the long (L’) and short (S’) alleles [43], exhibit distinct effects on gene expression and protein function. In vitro studies have demonstrated that cells homozygous for the L’ allele (L/L) synthesise significantly more serotonin transporter messenger RNA (mRNA)—approximately 1.4–1.7 times more—compared with cells with the S’ allele (S/L and S/S genotypes) [44]. Furthermore, the L/L variant displays a threefold higher basal activity of the 5-HTT protein and a 1.9–2.2 times greater capacity for serotonin reuptake from the synaptic cleft compared with the S/S and S/L variants [44]. These differences in transcriptional activity and protein function directly impact the efficiency of serotonin reuptake, a key mechanism in regulating serotonergic signalling.

Meta-analytic evidence suggests an association between genetic variation in the *SLC6A4* gene and the risk of AD. A comprehensive meta-analysis encompassing 22 studies (n = 8050) revealed the association between the possession of the S’ allele of the *5-HTTLPR* polymorphism and alcohol dependence [45]. This association was more pronounced in individuals with the S/S or S/L genotypes than in those with the L/L genotypes. Furthermore, the meta-analysis indicated that individuals diagnosed with alcohol dependence had a 15% greater likelihood of carrying at least one copy of the S’ allele. Earlier meta-analysis of 17 studies [46] also reported the association between the S’ allele and AD, with an odds ratio of 1.18 for the presence of the S’ allele in individuals with AD. Contrary to findings suggesting an increased risk of AD associated with the S’ allele, a study conducted in Korea [47] reported that individuals with L’ alleles had a higher risk of developing AD. This study also demonstrated a gene dose effect, with the “long” homozygous genotype associated with the highest risk, and found a higher frequency of the L allele in those with a family history of AD [47]. A Portuguese study, however, found a different distribution, with the L/L genotype being the most prevalent [48]. Some studies have identified sex-specific effects, finding an association [49] between the L/L genotype and severe alcohol dependence in women, reporting an association [50] between the L/L genotype and increased alcohol consumption in German adolescent females, and showing an association [51] between the L/L genotype and a higher incidence of AD in male subjects. Despite evidence suggesting a relation between the *5-HTTLPR* variants and AD, some studies have failed to find such a link. For example, no significant association was found between the *5-HTTLPR* variants and AD in a meta-analysis of 25 case-control studies [52]. Similarly, a German [53] and a Spanish case-control study [54] found no significant associations between the 5-*HTTLPR* variants and AD. Furthermore, Rasmussen et al. [55] also did not find an association in a study of older women with a history of severe AD, and Stoltenberg et al. [56] found no association in a family-based study of males, examining both the diagnosis of alcoholism and its severity. These conflicting results underscore the need for further research to elucidate the role of the 5-*HTTLPR* polymorphic variants in the development of alcohol use disorders.

The Five-Factor Model (FFM) represents a broadly accepted taxonomy for understanding and organising personality traits. Its development stemmed from a lexical approach and was refined through questionnaires and statistical techniques [57,58]. The FFM posits that personality can be comprehensively described by five broad traits: Extraversion, Agreeableness, Conscientiousness, Neuroticism, and Openness [59]. Empirical evidence supports the cross-cultural validity of the FFM, with translated measures demonstrating the relevance of these traits across diverse linguistic and cultural contexts [60,61]. Some researchers propose that these five traits reflect fundamental dimensions of human personality shaped by biological and evolutionary roots [62,63].

The NEO Personality Inventory Revised (NEO-PI-R) is a prominent tool for assessing the Five-Factor Model [64]. Current diagnostic approaches increasingly emphasise the assessment of dysfunctional personality traits, corresponding to extremes on the FFM dimensions, rather than relying solely on categorical diagnoses of personality disorders [4,65,66,67]. Research indicates that individuals with substance use disorders (SUD) exhibit a distinct FFM profile, characterised by elevated Neuroticism and reduced Conscientiousness and Agreeableness [68,69,70]. Higher Neuroticism scores have been associated with a range of psychiatric conditions [69], and this trait encompasses the propensity to experience negative emotions such as worry and anxiety [58]. It is hypothesised that those with higher Neuroticism may be more vulnerable to developing psychiatric disorders and that the psychological distress associated with substance use can further exacerbate Neuroticism. Lower levels of Conscientiousness, a trait reflecting organisation and planning [58], are associated with an increased risk of psychiatric disorders [69]. It is possible that individuals with low Conscientiousness are more susceptible to developing psychiatric disorders in response to stress or that psychological distress impairs their ability to plan and organise their lives effectively. Furthermore, Conscientiousness shares variance with executive functions, which are impaired in SUD [71], raising questions about their unique contributions to health outcomes [72]. Lower levels of Agreeableness, reflecting interpersonal tendencies toward altruism and trust [57], appear to be specifically associated with SUD but not with other psychiatric disorders [69]. Additionally, low Agreeableness may result from a history of childhood trauma [73] or comorbid ADHD [74], both of which can impair the ability to form healthy relationships.

The Spielberger State-Trait Anxiety Inventory (STAI) is a broadly used self-report questionnaire composed of 40 items designed to independently measure state anxiety and trait anxiety [75]. The STAI differentiates between state anxiety, conceptualised as a transient emotional state influenced by immediate situational factors, and trait anxiety, defined as a more enduring personality characteristic reflecting a general predisposition to experiencing anxiety. Anxiety is a commonly reported comorbidity in individuals diagnosed with AUD—a direct relationship between the intensity of anxiety symptoms and the degree of AD has been observed [76]. Anxiety is recognised as a considerable predictor of relapse among individuals undergoing treatment for AUD [76,77]. It is also noteworthy that anxiety is frequently associated with impaired emotional regulation, a factor that may contribute to the maintenance or exacerbation of AUD [78].

The main aim of this study was to analyse the association of the *5-HTTLPR* polymorphism and alcohol use disorder in a group of women and to perform an interaction analysis of 5-*HTTLPR* variants, personality traits, and alcohol use disorder.

## 2. Materials and Methods

### 2.1. Study Participants

The study group included 213 female volunteers, of which 101 were diagnosed with alcohol use disorder (AUD) (mean age = 45.74, SD = 11.11), and 112 were not dependent on any substance or behaviour (mean age = 45.32, SD = 10.19). The participants were Polish of Caucasian origin. Women with AUD were recruited from a single addiction rehabilitation centre. Inclusion criteria for the study were diagnosis of addiction according to DMS-V/ICD-10 criteria. Patients were recruited with 1–3 months of abstinence. The condition for admission to the rehabilitation centre was abstinence, measured with a breathalyser. A doctor assessed the patients’ mental state. Co-occurring mental disorders were a factor excluded from the study. The volunteers with AUD were not receiving treatment at the time of study participation. The control group comprised volunteers without AUD, without other comorbidities and were complete abstainers from alcohol. The controls were randomly sampled from the general population with an established screening tool—the AUDIT test (Alcohol Use Disorders Identification Test developed by the World Health Organization), to ensure that only people without alcohol addiction were recruited. The study group has been described in detail previously [79].

All participants (both the AUD and control groups) were voluntarily examined by a psychiatrist using the mini international neuropsychiatric interview (MINI), state-trait anxiety inventory (STAI), and NEO five-factor personality inventory (NEO-FFI) [79].

### 2.2. Genotyping

The polymorphism in the *SLC6A4* serotonin transporter gene promoter (5-*HTTLPR*) was identified by PCR with the pair primers: forward 5′-GGC GTT GCC GCT CTG AAT GC-3′ and reverse 5′-GAG GGA CTG AGC TGG ACA ACC AC-3′. The PCR products were for the long (L) allele, a 528 bp fragment, and the short (S) allele, a 484 bp fragment [41].

DNA samples (40 ng) were amplified in a final volume of 20 µL containing 4 pM of forward and reverse primer, 1x PCR Master Mix with polymerase Taq [Thermo Scientific™, Vilnius, Lithuania], and nuclease-free water. Amplification was performed using a VeritiPro 96-Well Thermal Cycler [Applied Biosystems^TM^ by Thermo Fisher Scientific^TM^, Singapore] under the following conditions: initial denaturation (95 °C/5 min.), followed by 38 cycles: denaturation (95 °C/30 s.), annealing (69 °C/60 s.), and elongation (72 °C/60 s.). PCR was terminated by 10 min of elongation at 72 °C. The resulting PCR products were separated electrophoretically in a 2% agarose gel stained with Midori Green DNA Stain [NIPPON Genetics Europe GmbH, Düren, Germany].

### 2.3. Statistical Analysis

The concordance between Hardy–Weinberg equilibrium (HWE) and the genotype frequency distribution was examined using the HWE software (https://wpcalc.com/en/equilibrium-hardy-weinberg/ online tool accessed on 5 April 2023).

The chi-square test was applied to conduct a correlation analysis between the *5HTT* (*SLC6A4*) variants and alcohol use disorder.

Levene’s test (*p* > 0.05) indicated that the condition of homogeneity of variance was met. The variables under analysis were not distributed normally. The Mann–Whitney U test was used to compare the scores of the STAI (Trait, State) and NEO Five-Factor Inventory (Neuroticism, Extraversion, Openness, Agreeability, and Conscientiousness) between cases and controls.

The relationship association between the *5HTT (SLC6A4*) variants, personality traits, and dependency was examined using a multivariate analysis of factor effects (ANOVA). The analysis used genotyping data from the AUD and control subjects, the STAI scales, and the NEO Five-Factor Inventory traits. The analysis included the following variables: genetic feature, STAI/NEO-FFI scale, control, and AUD (personality scale × genetic feature × control and AUD). The condition for homogeneity of variance has been met (Levene’s test *p* > 0.05).

The Bonferroni multiple comparisons correction was applied for these variables, and the accepted significance level was 0.0071 (0.05/7). All calculations were performed using STATISTICA 13 (Tibco Software Inc., Palo Alto, CA, USA) for Windows (Microsoft Corporation, Redmond, WA, USA).

## 3. Results

The frequency distribution of the *5HTTLPR* polymorphism genotypes was consistent with the HWE in the control subjects. In contrast, in AUD, there was a discrepancy with the HWE (Table 1).

We observe significant variations in the *5HTTLPR* genotypes (L/L 0.37 vs. L/L 0.49; S/S 0.27 vs. S/S 0.12; L/S 0.37 vs. L/S 0.38, χ^2^ = 7.545, *p* = 0.0230) and alleles (L 0.55 vs. L 0.68; S 0.45 vs. S 0.32, χ^2^ = 8.036, *p* = 0.0046) distribution when comparing the AUD group to the controls. Results are presented in Table 2.

In our previous study, we analysed the results of the NEO-FFI and STAI questionaries in AUD and case groups [79]. We observed significant differences in the mean scores of the analysed groups—the AUD subjects scored significantly higher on the anxiety state scales and anxiety trait. In addition, AUD group scores were significantly higher for the NEO-FFI Neuroticism and Openness scales. In the present study, we combined the previously obtained results from the STAI and the NEO-FFI with the *5-HTTLPR* genotyping data to perform the factorial ANOVA.

We observed a statistically significant impact of the AUD and control subjects on the STAI-T scale (F_1,207_ = 50.50; *p* < 0.0001; ɳ^2^ = 0.196;), on the STAI-S scale (F_1,207_ = 14.35; *p* = 0.0002; ɳ^2^ = 0.065), on the Neuroticism scale (F_1,207_ = 71.28; *p* < 0.0001; ɳ^2^ = 0. 256), on the Extraversion scale (F_1,207_ = 24.74; *p* < 0.0001; ɳ^2^ = 0.107), on the Openness scale (F_1,207_ = 5.09; *p* = 0.0250; ɳ^2^ = 0.024), on the Agreeability scale (F_1,207_ = 32.06; *p* < 0.0001; ɳ^2^ = 0.134), and on the Conscientiousness scale (F_1,207_ = 27.98; *p* < 0.0001; ɳ^2^ = 0.119).

We found a statistically significant impact of the *5HTT (SLC6A4)* genotype (F_2,207_ = 9.88; *p* = 0.0001; ɳ^2^ = 0.087; Table 3) on the Neuroticism scale. The power observed for this factor was 98%, and approximately 9% was explained by the polymorphism of the *5-HTT (SLC6A4)* gene on the trait’s score variance.

Additionally, we observed a statistically significant impact of the *5-HTT* (*SLC6A4*) genotype (F_2,207_ = 5.74; *p* = 0.0037; ɳ^2^ = 0.053; Table 3) on the Extraversion scale. The power observed for this factor was 86%, and approximately 5% was explained by the polymorphism of the *5HTT* (*SLC6A4*) on the trait’s score variance.

**Figure 1:**
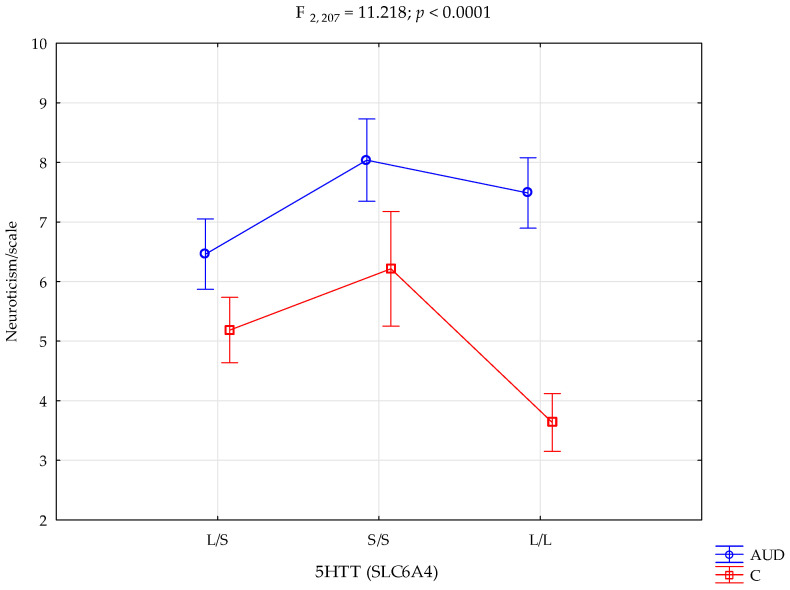
Interaction between the alcohol use disorder group (AUD), controls (C), *5HTT (SLC6A4)*, and the NEO-FFI Neuroticism scale.

In addition, there was a statistically significant impact of *5HTT (SLC6A4)* genotype interaction and alcohol addiction or lack of it on the Neuroticism scale (F_2,207_ = 11.22; *p <* 0.0001; η^2^ = 0.098; Table 3, Figure 1). The power observed for this factor was 99%, and approximately 10% was explained by the polymorphism of the *5HTT (SLC6A4)* genotype and alcohol addiction or lack thereof on the Neuroticism trait score variance.

The results of the factorial ANOVA of the State-Trait Anxiety Inventory, NEO Five-Factor Inventory traits, *5HTT (SLC6A4)*, AUD, and controls are presented in Table 3.

Performed post hoc analysis showed that AUD subjects with the *5HTT (SLC6A4)* L/L and S/S genotypes obtained significantly higher results on the NEO-FFI Neuroticism scale than control subjects with the L/S, L/L, and S/S genotypes. In addition, the AUD participants with the L/S genotype obtained significantly higher results on the Neuroticism scale than the controls with the L/S and L/L genotypes. Unlike above, the AUD subjects with the L/S genotype obtained significantly lower results on the Neuroticism scale than AUD subjects with the S/S and L/L genotypes. Controls with the L/L genotype obtained significantly lower Neuroticism scores than control subjects with the L/S and S/S genotypes. Table 4 presents the results of the post hoc test.

## 4. Discussion

The key topic of our research is the study of the correlations between genetic factors and personality traits of women with AUD. The previous results indicate that individuals with AUD are more likely to exhibit Neuroticism and Anxiety-related traits at the high sten level and Openness, than Anxiety-related traits in the medium sten score. When comparing the two groups, women without AUD express more Extraversion and Agreeableness behaviours at the moderate level, indicating more introverted and mistrustful behaviours among individuals with AUD [79]. This may indicate the effect of excessive alcohol consumption among women on behavioural changes compared with non-addicted women. Greater sensitivity to fear and stress associated with specific situations and emotional imbalance tilted toward negative reactions in women who consume alcohol may suggest a greater likelihood of behaviours associated with general depression, which could indirectly indicate the existence of the AUD phenotype in women [80,81]. A meta-analysis summarizing the results of 38 studies suggests the existence of two personality subtypes found in people with AUD. The first type is an impulsive, extroverted, and experience-seeking personality and the second subtype is an anxiety and introverted behaviour personality. This analysis indicates the complexity of the problem of determining the phenotype of a person dependent on alcohol through the influence of modulating genetic factors including, in particular, neurobiological markers. At the same time, it suggests that some personality traits may be related with the occurrence of alcohol disorder [82].

In this study, the main claim describes the relationship between personality features and the *5-HTTLPR* genotype variants in women of Caucasian origin with confirmed alcohol dependence. This study involved 213 women, including 101 with confirmed AUD and 112 women who were free of addiction. For this analysis, genotypes of *5-HTTLPR*, along with personality traits defined by NEO-FFI and anxiety levels described in STAI scores, were determined. In addition, the correlations between AUD, the 5-HT transporter-linked promoter region polymorphism, and personality traits were determined.

The limitation of our study was the lack of sociodemographic data (including family history, quality of life, occupation, or education) that could have enriched our analyses of indirect effects on the occurrence of alcohol disorders. The literature indicates the potential impacts of these factors on the risk of AUD among the subjects. An example is a study from Singapore, which indicates the existence of differences between occupational groups, including differences in education and the increase or decrease in the risk of addiction among people [83]. Previous studies have found that differences in the quality of life (QoL) between individuals with alcohol use disorders and control individuals who did not consume alcohol were statistically significant in the domains of physical health, psychological health, and social relationships. With regard to quality of life, a significant relationship between marital status and the domain of social relations has been demonstrated, including a particularly positive effect of marriage among people with AUD [84]. Other studies also confirm this observation, which indicates a beneficial effect of co-accommodation with a partner on the social aspects of quality of life [85,86]. In the context of assessing the various domains of quality of life, some studies suggest that modulators such as education and age do not affect them [85]. However, other studies suggest opposing observations, showing evidence of the negative impact of lower levels of education and older age on people’s quality of life [87,88]. Another modulating factor is the family history of addiction. Research indicates that there is an indirect effect on the inheritance of predisposition in families with a history of addiction. Indirect family influence may result from the occurrence of stressful situations resulting from alcohol abuse at home, such as the divorce of the parents [89]. Additionally, there are studies indicating that a positive family history not only increases environmental risk but can also complement polygenetic risk assessment [90]. Demographics, as described above, may indirectly explain the risk of AUD. Supplementing our results with this information could potentially correct the influence of genetic background on the prevalence of addiction among the women included in this study. This is a limitation that must be taken into account when interpreting our results.

Epidemiological studies indicate differences in the incidence and intensity of various mental illnesses between men and women, such as anxiety disorders, substance and alcohol abuse, and antisocial anxiety disorders [91]. Additionally, it has been suggested that women are more likely to exhibit behaviours related to neuroticism and anxiety; compared with men, more openness to experience and self-confidence are observed. Studies observing brain function and structure indicate physiological differences between the sexes in the interpretation of processed data, such as differences in the number of receptors and cell type [92]. Women are more likely to exhibit disorders related to emotional imbalance, such as major depressive disorder (MDD), which is accompanied by lower concentrations of 5-HT, and men more often exhibit neuropsychiatric disorders, such as schizophrenia with higher serotonin production [93]. So far, researchers have identified several reasons for the differences in serotonin metabolism between the sexes. One of them is the interaction between gonadal steroids and the serotonergic system. Estrogen receptors are located in key areas of the brain, including the hypothalamus and amygdala, and are responsible for mood. Estrogen itself regulates the activity of the serotonin transporter and reduces the activity of 5-HT breakdown enzymes (MAO-A and MAO-B). At the same time, it may stimulate the work of tryptophan hydroxylase, resulting in increased serotonin synthesis [91]. Another suggested cause may be a difference in the metabolism of tryptophan in 5-HT. Some animal studies indicate a significantly higher percentage of circulating tryptophan and, thus, 5-HT synthesis in females, which has also been noted in clinical data observed in women. Other reports indicate an increase in the activity of the serotonergic system (which suggests a higher metabolic rate of serotonin in the brain) in healthy women compared with men. In addition, gonadal steroids such as estrogen also affect the amount of circulating tryptophan, which indirectly moderates the synthesis of 5-HT. Differences in the concentrations of these hormones during the menstrual cycle in women may contribute to the formation of differences in the susceptibility and phenotypes of various neuropsychological disorders against the background of disorders of tryptophan metabolism between the sexes [93].

The above-mentioned examples suggest the existence of gender-directional tendencies in relation to emotional and mental state on the basis of the serotonergic system. For this reason, only women were included in our study to illustrate the differences in the intensity of individual personality traits in the context of the occurrence/absence of AUD and the differences in *5-HTTLPR* variants, without interference from the different physiological state of serotonin metabolism in men. These differences could hypothetically shift interpretations regarding the correlation between the occurrence of a specific genotype and the occurrence of a specific personality trait among addicted and non-addicted people. In future studies, we will expand the study group to include a cohort of men to test the effect of different sexes on correlation results. In addition, we limited our study group to only people of Caucasian descent. Studies on various conditions, such as the level of behavioural anxiety and neurophysiological indicators in maintaining executive control [94], PTSD [95], or depression [96], indicate differences in the distribution of allele variants depending on ethnicity and race. In order to reduce disruptions in the distribution of alleles, we applied our results to a group of homogeneous ethnicity.

In the next stage of this study, the prevalence of VNTR *5-HTTLPR* polymorphism (L/L, L/S, S/S) alleles was obtained by comparing the groups. In our study, a significant increase in the percentage of S’ alleles among women suffering from AUD compared with the control was observed, while maintaining similar results for genotypes with the L’ allele. This suggests a delicate relationship between the prevalence of addiction and the genotype associated with short allele repetition. So far, there have been many conflicting analyses regarding the extent to which each variation in this polymorphism affects the potential for excessive alcohol consumption. Some studies indicate that the occurrence of L/L homozygotes is associated with the presence of increased sensitivity to a limited response to alcohol, which indirectly contributes to the development of AUD in subjects. Additionally, the L/L genotype has been shown to be connected with increased early alcohol consumption by young people in a group of 72 participants (male = 47, female = 25) [97]. On the other hand, researchers have also shown a positive association between alcoholism and a person’s possession of the S/S genotype (n = 130, male = 118, female = 12) [98]. A meta-analysis from 2005 (including 17 studies) found that the S’ allele is more common among people with AUD than the L’ allele. Numerous studies have shown a positive correlation between the occurrence of a short gene variant and an increased susceptibility to negative alcohol-related behaviours such as binge drinking, higher frequency of occasional drinking, and drinking with the intention of getting drunk [99,100]. Another meta-analysis including 22 studies implied that people with AUD are approximately 15% more likely to have the S’ allele as a factor influencing the onset of addiction. Nevertheless, the outcomes of these studies should be considered with caution due to the statistical significance at the acceptable boundary, as the authors of the publication themselves highlight [45]. One recent analysis suggests a correlation between the appearance of alleles associated with low serotonergic response in the body in the 5-HTT transported polymorphism and SNP rs25531. The study had two groups: one with 1447 participants with AUD (male = 1071, female = 376) and the second with 441 participants without AUD as a control. Among individuals with diagnosed AUD, a significant increase in the proportion of genotypes with S’ and L_G_ alleles was observed [101].

Additionally, in this study, a 10% indirect effect of genotype *5-HTTLPR* was correlated with a larger prevalence of neurotic-related traits in women with alcohol addiction in comparison with the women without alcohol addiction (strength of the factor was 99%). Both the L/L and S/S genotypes achieved a higher level of correlation with the occurrence of this trait in combination with alcohol problems (S/S 8.04 ± 1.91, L/L 7.49± 1.79, respectively). However, S/S homozygotes scored higher on the sten scores, suggesting a greater importance of the S’ allele in contributing to neuroticism traits in addiction compared with L/L. This may be hypothesized to link more depression- and anxiety-related behaviours in women who consume alcohol.

At the same time, the difference in the number of points on the sten scale between L/L homozygotes with AUD and the controls (7.49± 1.79, 3.64 ± 1.95) was significantly greater than in the case of S/S homozygotes (8.04 ± 1.91, 6.21 ± 2.08). Women without alcohol use disorders scored lower Neuroticism sten scores for the long allele. Hypothetically, this may suggest the existence of protective properties of the L’ allele against the tendency to behaviours contributing to depression among individuals without addiction compared with addicted women. Previous studies indicated that L/L homozygotes in alcohol dependence contribute to violence over the course of an addiction with higher symptoms of depressive behaviour, suggesting a combined sympathology of depression in combination with AUD [97]. Similar differences between groups regarding the L/L genotype can be seen at the level of the STAI-T and STAI-S traits. The difference between alcoholics and women without AUD was greater on the sten scale in the presence of L/L homozygotes. However, a 2000 study suggested that long-term alcohol use causes low 5-HTT binding in individuals with the L/L genotype [102]. Differences in serotonin neurotransmission through the indirect effect of alcohol in the presence of different polymorphism variants may be the basis for understanding the differences in the intensity of the above-mentioned traits between the AUD group and the controls.

Studies in recent years have also focussed on the association of *5-HTTLPR* polymorphism with behavioural changes, including in people with alcohol problems. According to a study, the short allele was more likely to be detected in young people who developed a problem with excessive alcohol consumption after experiencing negative life events [97,103]. Behaviours leading to the development of depression, such as stress sensitivity and neuroticism, were also associated with the S/S genotype (n = 50, female = 47) [104]. In addition, an analysis of behaviours leading to depression found an increase in traits such as impulsivity, hostility, neuroticism, and anxiety traits in homozygous S/S people with a history of family depression (n = 203, female = 54%) [105]. Reduced transcription activity associated with carrying the S’ allele may hypothetically induce a greater response from the amygdala. Consequently, this leads to the occurrence of traits related to anxiety or depressive behaviours [106]. Over the years, many studies have linked the incidence of the S’ allele-homozygous with significant neurotic scores, as well as anxiety as a trait and higher levels of emotional reactivity in personality and trait tests [103,107]. Such a trait picture may be related to serotonin’s role in growth processes and neurite modelling in which variants of the 5-HTT polymorphism may affect developmental mechanisms and synaptic signal transduction in adults. These studies confirm that there is a reduced flow of stimuli between the pre-frontal cortex and the amygdala with a short *5-HTTLPR* allele. This indicates an overall decreased functionality between the indicated brain regions [103,108]. A 2008 meta-analysis grouping study related to serotonin transporter polymorphism with changes in amygdala activity confirmed that serotonin transcription variability influences behavioural changes. The analysis suggests that approximately 10% of phenotype variations associated with anxiety and mood disorders are caused by *5-HTTLPR* via amygdala–body activation. However, an association with a specific allele variant has been suggested as a factor in moderating ethnicity as an influence of genetic background [109]. It has also been suggested that the short 5-HTT allele is associated with greater amygdala activity in response to stimuli. In addition, it was discovered that the same allele may be responsible for the reduction in grey matter within the amygdala and the rACC/vmPFC region (the rostral anterior cingulate cortex/the ventromedial prefrontal cortex, respectively) and the weakening of the functional and morphological link of both structures, resulting in a decrease in the control of negative emotions expression [110]. Studies on the understanding of suicide attempts in depression among men have noted a relationship between the occurrence of the S’ allele and lower availability of 5-HTT in the frontal cortex, which confirms previous reports of reduced transporter amounts in the hypothalamus, PFCs, and brainstem. This indicates the existence of the influence of the *5-HTTLPR* polymorphism on the serotonergic system among psychiatric disorders [111]. The effect of changes in the serotonin transporter polymorphism on 5-HT neurotransmission may have various causes. To gain a comprehensive insight into the correlation between *5-HTTLPR* and various behavioural and neuropsychiatric disorders, serotonin concentrations in the body can be included in the study. Such an approach would provide a broader context on the indirect effect of a gene on an organism. Unfortunately, our study does not include data on serotonin concentrations. Only the distribution of alleles in the context of *5-HTTLPR* has been developed.

The average serotonin level among alcoholics would theoretically be expected to be higher compared with the control group due to the higher prevalence of S’ alleles and, thus, reduced serotonin reuptake. As mentioned earlier in this article, research suggests that alcohol may indirectly contribute to an increase in serotonin levels. For this reason, hypothetically, the difference in concentrations between the groups should be clearly marked by combining the effect of alcohol consumption and the effect of allele frequency. However, the occurrence of clear neuroticism in the AUD group in women with the L/L and S/S genotypes indicates potential inaccuracies in the level of serotonin concentration under the influence of *SLC6A4* and the occurrence of traits associated with depressive behaviours. It should also be noted that there are other factors besides the *5-HTTLPR* polymorphism that can affect the concentration of serotonin in the body. Without obtaining results from serotonin levels among women without AUD as well as those with AUD, we can only speculate how this information could affect the analysis of our results in the context of the presence of these characteristics. This is another limitation compared with our results. In subsequent tests, we will deepen the diagnostics by measuring the amount of serotonin in the body.

As we presented earlier, the short allele of the *SlC6A4* polymorphism influences, to some extent, the intensification of the traits associated with neuroticism, especially among women with AUD. At the same time, in our study, a generally higher Neuroticism score can be observed among the control group with the S/S genotype individuals (6.21 ± 2.08). We confirmed that polymorphisms *5-HTTLPR* in the context of alcohol dependence can explain the 10% incidence of intensification of these traits in women. Among participants without AUD or AD, it is likely that other external factors may have contributed, due to the multifactorial background behind the occurrence of depression-related traits. The S’ allele itself has previously been linked to the occurrence of behaviours such as feelings of hopelessness, anxiety, neurotic disorders, and other components of neuroticism among healthy individuals [112]. Over the years, various factors such as stressful life situations have been studied, which can likely also modulate the interaction between S/S healthy individuals and tendencies in psychopathological processes [113,114]. This has been shown, among others, in a study on adolescents on attention bias to negative emotions, *5-HTTLPR*, and recent stress exposure [115], and in a study of a group of women on the effect of rapid exposure to stress versus the reduction in inhibitory effects on the processing of negative information [116]. Researchers also highlight other factors modulating the influence of S/S on depression-related traits in healthy individuals, such as: relational insecurity in youth [117] and positive familial burden of depression [105]. Reports over the years indicate that environmental influences may significantly modulate the occurrence of traits in healthy individuals with the S/S genotype, which could explain the high Neuroticism sten scores in the control group in our study.

Some researchers also indicate that the L’ allele is related to depressive, impulsive, and compulsive–obsessive behaviours, including compulsive alcohol consumption [98]. A 2011 study indicated a significant impact of the occurrence of the L’ variant in a group of 72 (male = 47) individuals with increased alcohol problems, along with the appearance of depressive traits examined using the BDI-II questionnaire [97]. A study on a group of 91 American veterans (men = 82, women = 9) undergoing treatment for addiction also did not find a significant connection between symptoms of depression-related traits and the occurrence of homozygous S/S. However, this analysis had many limitations that should be considered [118].

For many years, *5-HTTLPR* polymorphism has been studied in terms of its indirect effect on the development of depression in people when taking into account stressful life situations. A meta-analysis of 54 studies from 2011 confirmed the significant impact of polymorphism variants (S’ allele) as a moderator of the correlation between stress (as specific diseases, childhood maltreatment) and the occurrence of depression over the course of a life. However, the direct relationship with stressful life situations was marginally significant [114]. However, another meta-analysis of 47 studies from 2015 on the risk of developing depression among young adults and adolescents confirmed the indirect effect of *5-HTTLPR* in adverse life experiences [119]. These two big analyses suggest that there are a number of factors (stressors) that may contribute to the correlation between the occurrence of depression and the presence of variants of the serotonin transporter promoter polymorphism. Our study only considered alcoholism as an environmental factor affecting emotional state in the context of the occurrence of different variants of *5-HTTLPR.* Because of the existence of diverse stressors in the genetic ethology of depression, this might be considered a limitation of our study in the context of the specificity of our results.

The differences between the occurrence of traits such as neuroticism, anxiety, or impulsiveness and the genotype of polymorphism can have different backgrounds. One of the moderating factors identified in the literature is gender. A study based on an analysis of sociopathy in people with AUD highlighted the strong influence of gender on the results. The study group consisted of 862 participants, of which 73.4% were men, of mostly Caucasian origin. In women, traits associated with sociopathy were associated with genotype S/S, whereas the opposite results were obtained in the group of exclusively men with genotype L/L [100]. Two studies conducted by Brummett et al. confirmed the difference in results by sex differences. In the first case, involving 288 people (women = 215, men = 73), the role of polymorphism in relation to the onset of caring stress (people living in a continuous stressful situation) was examined, while in the second case, involving 142 people (women = 64, men = 78), the association with stress caused by low socioeconomic status in childhood was examined. In both cases, a significant correlation between increased symptoms of depression caused by different types of stressful situations was found with the S/S homozygotes variant in women and L/L in men [120]. Another study showed the importance of the impact of gender on the association of SLC6A4 polymorphism and mental state is a cohort study of 2236 Han Chinese people. In this experiment, a group of patients with diagnosed generalized anxiety syndrome (GAD) (n = 736, male = 315, female = 421) and a group of individuals without GAD (n = 1500, male = 730, female = 770) were examined in terms of genotypes and Chinese equivalents of the Maudsley Personality Inventory (MPI-Neuroticism) and the Beck Anxiety Inventory (BAI) were examined. The results indicated that women with the S/S variant scored higher in MPI-Neuroticism, whereas men showed an inverse correlation by obtaining similar results with the L’/L’. The increased risk of GAD was confirmed and the increased level of neuroticism in the presence of a given genotype was found only among the group of women [121]. There are also studies suggesting that gender is not relevant to the prevalence of traits associated with depression or anxiety. A 2000 study, complementing an earlier cohort of women (a total of 532 men and 370 women), compared the results of personality tests and the prevalence of *5-HTTLPR* genotypes. They showed no differences between the two studies. Both women and men showed increased neuroticism and reduced levels of agreeability with the presence of the S’ allele [122]. The research indicates the significant effect of gender in estimating the connection between SLC6A4 variants and symptoms associated with depression or anxiety, which may, to some extent, explain the additional results linking these traits with the S/S genotype in AUD women as an effect of gender as a moderator of neurotic intensity.

As the above examples from the literature show, gender is an important modernizing factor in the correlation between *5-HTTLPR* and the occurrence of individual personality traits. Most studies analysing personality traits and the polymorphism of the serotonin transporter promoter in various diseases are based on mixed groups of subjects, with males largely making up the majority of the groups. Alcoholism, as previously emphasized, has recently been studied for this polymorphism, and some researchers also describe the effect on behavioural changes. However, the modernizing factor that is gender is rarely taken into account, and mixed groups are analysed as a whole.

The links between polymorphism itself and personality traits in a group of healthy women have previously been investigated under different conditions. An example of this study is an experiment analysing the responses of the amygdala to negative affective stimuli in the presence of various *5-HTTLPR* genotypes among a group of 55 Korean women. They highlighted the significant impact of the L’ allele on the activity of the amygdala when women were exposed to negative facial stimuli [123]. Another example, analysing only women, was a study on the correlation between personality traits, *5-HTTLPR,* and the occurrence of premenstrual dysphoric disorder (PMDD). This study showed that the short allele has an effect on increased anxiety-related traits in women with PMDD and women without PMDD [124]. Analysis of a group of 273 postmenopausal women from Poland considering traits related to anxiety, on the other hand, showed no significant impact of *5-HTTLPR* polymorphism on mood and anxiety-related behaviours [125].

There are few studies looking at the impact of alcohol use disorders on sex, although, as previously described, there is proof for the overall effect of *SLC6A4* genotypes on behavioural changes among addicts. There is a limited number of studies looking at the effect of polymorphism and AUD alone in women.

A similar study on alcoholism in women from 2008 examined the association between *SLC6A4* polymorphism and monoamine oxidase A promoter polymorphism (MAOA-VNTR) and severe cases of alcoholism in women. This study did not focus on analysing the effect of changes on behaviours among the study group but only on the prevalence of alleles, although the researchers divided the women into two subgroups: alcoholics with and without co-existing psychiatric disorders. They discovered a significantly higher frequency of L/L variants in women with severe AUD when excluding participants with diagnosed psychological disorders [49]. Another study focussing on the presence of alleles in alcoholic women involved 1365 older women from Denmark. The main aim of the study was to observe a connection in the SLC6A4 polymorphism and the frequency of current/past smoking and drinking in older women. However, the researchers did not show any effect of either genotype on the frequency of drinking or lack of alcohol consumption [55]. These two publications indicate that there has been interest among researchers in understanding the prevalence of addiction in women with a view to analysing this polymorphism.

However, to our knowledge, we have not found any similar studies that have focussed on the impact of *SLC6A4* polymorphism on the occurrence of various personality traits between women with AUD and women without AUD. We did not observe any studies that have directly focussed on the differences in personality traits depending on the occurrence of AUD or the absence of an allele *5-HTTLPR* in women when searching the PubMed database using keywords such as: “5-HTTLPR”, “women”, “personality traits”, “alcohol addiction”, “SLC6A4”, “AUD”, “NEO-FFI”, and the like. This indicates that our study is the first to report results directly on this correlation. The association of behaviours associated with depression, including neuroticism, in women with AUD and a specific variant of the *5-HTTLPR* is another factor enabling the understanding of the mechanism of addiction, including the different effect of gender, as well as the differentiation of alcoholic phenotypes among women. The lack of previous research in this direction allows for greater possibilities to develop this topic in the future for a more in-depth analysis of this correlation with new factors.

In conclusion, our study showed an important distinction between personality traits between women without AUD and AUD sufferers in terms of negative emotional responses (neuroticism), state anxiety, and traits that indicate the existence of a phenotype among alcoholics. By expanding this analysis (the first to analyse this link), we demonstrated the significant impact of high levels of 5-HTTLPR polymorphism on symptoms of neuroticism between groups. The influence of genotype variability in the 5-HTT (SLC6A4) may partially explain the differences in the behaviour of alcohol-dependent women by affecting the serotonergic system.

## 5. Conclusions

This study investigated the relationship between *5-HTTLPR* polymorphism in the *SLC6A4* gene, personality traits, anxiety, and alcohol use disorder. Our findings reveal significant differences in the 5-*HTTLPR* genotype and allele distribution between individuals with AUD and controls, suggesting a potential association between this polymorphism and susceptibility to AUD. Specifically, we observed a higher proportion of the L/L genotype and L’ allele in the control group compared with the AUD group, while the S/S genotype and S’ allele were more prevalent in the AUD group.

Importantly, we observed a significant effect of the *5-HTTLPR* genotype on both Neuroticism and Extraversion scores. This suggests that the *SLC6A4* polymorphism may influence these personality traits, potentially through its role in serotonin neurotransmission. The extent of these effects, as indicated by the partial eta-squared values, suggests that the *SLC6A4* polymorphism accounts for a notable, albeit modest, proportion of the variance in Neuroticism (8.7%) and Extraversion (5.3%).

The main finding is the significant interaction between the *5-HTTLPR* genotype and AUD status on Neuroticism scores. This indicated that the association between the *5-HTTLPR* genotype and Neuroticism is not uniform across the AUD and control groups. The observed interaction suggested that the effect of the *5-HTTLPR* polymorphism on Neuroticism may be exacerbated or attenuated in the presence of AUD. Further investigation is needed to elucidate the precise nature of this interaction. Still, it potentially points to a gene–environment interaction where the genetic predisposition conferred by the *5-HTTLPR* polymorphism interacts with the environmental stressor of alcohol use disorder to influence Neuroticism. This interaction accounted for approximately 10% of the variance in Neuroticism scores, highlighting its potential clinical significance.

In conclusion, our findings support the hypothesis that the insertion/deletion polymorphism located in the promoter region of the *SLC6A4* gene is associated with both susceptibility to AUD and variation in personality dimensions, particularly Neuroticism and Extraversion. This study demonstrated a significant interaction between genotype and AUD status on Neuroticism. It highlighted the intricate interplay between genetic predisposition and environmental factors in shaping individual vulnerability to both psychiatric disorders and personality characteristics.

## Figures and Tables

**Table 1:** Hardy–Weinberg’s equilibrium of the *5HTTLPR* polymorphism for the AUD group and control subjects.

Hardy–Weinberg Equilibrium, Including Analysis for Ascertainment Bias		Observed (Expected)	Allele Freq	χ^2^ (*p*-Value)
AUD n = 101	L/S	37 (30.5)	p (L) = 0.55 q (S) = 0.45	6.831 (0.0089)
	S/S	27 (20.5)		
	L/L	37 (50.0)		
Control n = 112	L/S	43 (48.5)	p (L) = 0.68 q (S) = 0.32	1.438 (0.230)
	S/S	14 (11.3)		
	L/L	55 (52.3)		

*p*-value—statistical significance, χ^2^ test; AUD—alcohol use disorder.

**Table 2:** Frequency of genotypes and alleles of *5HTTLPR* polymorphisms in the alcohol use disorder and control subjects.

	Genotypes			Alleles	
	L/S	S/S	L/L	L	S
	n (%)	n (%)	n (%)	n (%)	n (%)
AUD	37	27	37	111	91
n = 101	(36.63%)	(26.73%)	(36.63%)	(54.95%)	(45.05%)
Control	43	14	55	153	71
n = 112	(38.39%)	(12.50%)	(49.11%)	(68.30%)	(31.70%)
χ^2^	7.545			8.036	
(*p*-value)	(0.0230)			(0.0046)	

n—number of subjects; *p*-value—statistical significance, χ^2^ test; AUD— alcohol use disorder.

**Table 3:** The 2 × 3 factorial ANOVA results for AUD and control subjects, NEO-FFI, STAI, and *5HTTLPR* genotypes.

STAI NEO-FFI	Group	*5HTT (SLC6A4)*				ANOVA		
		L/S n = 80 M ± SD	S/S n = 92 M ± SD	L/L n = 41 M ± SD	Factor	F (*p*-Value)	ɳ^2^	Power (alpha = 0.05)
STAI trait scale	Alcohol use disorder (AUD); n = 101	7.24 ± 2.28	7.93 ± 2.02	7.43 ± 2.25	Intercept AUD/control 5HTT (SLC6A4) AUD/control × *5HTT (SLC6A4)*	F_1,207_ = 1432.82 (*p* < 0.0001) *# F_1,207_ = 50.50 (*p* < 0.0001) *# F_2,207_ = 2.52 (*p* = 0.0833) F_2,207_ = 1.56 (*p* = 0.2121)	0.874 0.196 0.024 0.015	1.000 1.000 0.500 0.329
	Control; n = 112	5.33 ± 2.33	5.79 ± 2.19	4.35 ± 2.20				
STAI state scale	Alcohol use disorder (AUD); n = 101	5.68 ± 2.48	6.37 ± 2.69	6.11 ± 2.41	Intercept AUD/control 5HTT (SLC6A4) AUD/control × *5HTT (SLC6A4)*	F_1,207_ = 961.96 (*p* < 0.0001) * F_1,207_ = 14.35 (*p* = 0.0002) *# F_2,207_ = 1.17 (*p* = 0.3128) F_2,207_ = 2.00 (*p* = 0.1382)	0.823 0.065 0.011 0.019	1.000 0.965 0.255 0.410
	Control; n = 112	5.02 ± 1.91	5.14 ± 1.83	4.04 ± 2.31				
Neuroticism scale	Alcohol use disorder (AUD); n = 101	6.46 ± 1.61	8.04 ± 1.91	7.49 ± 1.79	Intercept AUD/control 5HTT (SLC6A4) AUD/control × *5HTT (SLC6A4)*	F_1,207_ = 2024.63 (*p* < 0.0001) *# F_1,207_ = 71.28 (*p* < 0.0001) *# F_2,207_ = 9.88 (*p* = 0.0001) *# F_2,207_ = 11.22 (*p* < 0.0001) *#	0.907 0.256 0.087 0.098	1.000 1.000 0.983 0.992
	Control; n = 112	5.19 ± 1.71	6.21 ± 2.08	3.64 ± 1.95				
Extraversion scale	Alcohol use disorder (AUD); n = 101	5.59 ± 2.07	4.00 ± 2.37	5.43 ± 2.09	intercept AUD/control 5HTT (SLC6A4) AUD/control × *5HTT (SLC6A4)*	F_1,207_ = 1452.24 (*p* < 0.0001) *# F_1,207_ = 24.74 (*p* < 0.0001) *# F_2,207_ = 5.74 (*p* = 0.0037) *# F_2,207_ = 2.04 (*p* = 0.1328)	0.875 0.107 0.053 0.019	1.000 0.999 0.863 0.417
	Control; n = 112	6.30 ± 1.67	6.00 ± 1.88	7.24 ± 1.99				
Openness scale	Alcohol use disorder (AUD); n = 101	4.81 ± 1.75	4.96 ± 2.41	5.51 ± 2.28	intercept AUD/control 5HTT (SLC6A4) AUD/control × *5HTT (SLC6A4)*	F_1,207_ = 1104.62 (*p* < 0.0001) *# F_1,207_ = 5.09 (*p* = 0.0250) * F_2,207_ = 1.00 (*p* = 0.3689) F_2,207_ = 0.65 (*p* = 0.5259)	0.842 0.024 0.009 0.006	1.000 0.613 0.223 0.157
	Control; n = 112	4.51 ± 1.72	4.29 ± 1.68	4.55 ± 1.63				
Agreeability scale	Alcohol use disorder (AUD); n = 101	3.89 ± 1.91	3.85 ± 2.03	3.78 ± 1.75	intercept AUD/control 5HTT (SLC6A4) AUD/control × *5HTT (SLC6A4)*	F_1,207_ = 945.82 (*p* < 0.0001) *# F_1,207_ = 32.06 (*p* < 0.0001) *# F_2,207_ = 1.09 (*p* = 0.3365) F_2,207_ = 1.53 (*p* = 0.2184)	0.820 0.134 0.010 0.015	1.000 0.999 0.241 0.324
	Control; n = 112	4.93 ± 1.93	6.00 ± 2.22	5.80 ± 2.30				
Conscientiousness scale	Alcohol use disorder (AUD); n = 101	5.00 ± 2.43	4.81 ± 2.24	5.05 ± 2.37	intercept AUD/control 5HTT (SLC6A4) AUD/control × *5HTT (SLC6A4)*	F_1,207_ = 1275.86 (*p* < 0.0001) *# F_1,207_ = 27.98 (*p* < 0.0001) *# F_2,207_ = 1.48 (*p* = 0.2299) F_2,207_ = 0.75 (*p* = 0.4751)	0.860 0.119 0.014 0.007	1.000 0.999 0.314 0.176
	Control; n = 112	6.60 ± 2.15	6.14 ± 1.66	7.29 ± 1.90				

*—significant result; AUD—alcohol use disorder; M ± SD—mean ± standard deviation. # Bonferroni correction was applied, and the *p*-value was lowered to 0.0071 (*p* = 0.05/7 (number of statistical tests performed)).

**Table 4:** Post hoc test (Least Significant Difference) of interactions between the alcohol use disorder group, controls, *5HTT (SLC6A4)*, and the NEO-FFI openness scale.

	{1}	{2}	{3}	{4}	{5}	{6}
	M = 6.46	M = 8.04	M = 7.49	M = 5.19	M = 6.21	M = 3.54
**AUD L/S {1}**		0.0008 *	0.0162 *	0.0021 *	0.6684	0.0000 *
**AUD S/S {2}**			0.2338	0.0000 *	0.0027 *	0.0000 *
**AUD L/L {3}**				0.0000 *	0.0271 *	0.0000 *
**Control L/S {4}**					0.0680	0.0000 *
**Control S/S {5}**						0.0000 *
**Control L/L {6}**						

*—significant statistical differences; M—mean.

## Data Availability

The genotyping and psychometric test results are available upon request.
